# Mercuric Compounds Induce Pancreatic Islets Dysfunction and Apoptosis *in Vivo*

**DOI:** 10.3390/ijms131012349

**Published:** 2012-09-26

**Authors:** Kuo-Liang Chen, Shing-Hwa Liu, Chin-Chuan Su, Cheng-Chieh Yen, Ching-Yao Yang, Kuan-I Lee, Feng-Cheng Tang, Ya-Wen Chen, Tien-Hui Lu, Yi-Chang Su, Chun-Fa Huang

**Affiliations:** 1 Department of Urology, China Medical University Hospital, and School of Medicine, China Medical University, No.2 Yuh-Der Rd., Taichung 404, Taiwan; E-Mail: ckl_2001@yahoo.com; 2 Institute of Toxicology, College of Medicine, National Taiwan University, No.1 Jen-Ai Rd., Section 1, Taipei 100, Taiwan; E-Mail: shliu@ha.mc.ntu.edu.tw; 3 Department of Otorhinolaryngology, Head and Neck Surgery, Changhua Christian Hospital, No.135 Nanxiao St. Changhua City, Changhua County 500, Taiwan; E-Mail: 91334@cch.org.tw; 4 Department of Occupational Safety and Health, College of Health Care and Management, Chung Shan Medical University; and Department of Occupational Medicine, Chung Shan Medical University Hospital, No. 110 Section 1, Jian-Guo N. Rd., Taichung 402, Taiwan; E-Mail: ycj@csmu.edu.tw; 5 Department of Surgery, National Taiwan University Hospital, and Department of Surgery, College of Medicine, National Taiwan University, Taipei 10043, Taiwan; E-Mail: cyang@ntuh.gov.tw; 6 Department of Emergency, Buddhist Tzu Chi General Hospital, Taichung Branch, No. 66 Section 1, Fongsing Rd., Tanzih Township, Taichung 427, Taiwan; E-Mail: leeguanto2002@yahoo.com.tw; 7 Department of Occupational Medicine, Changhua Christian Hospital, Changhua 500, Taiwan; E-Mail: 106159@cch.org.tw; 8 Department of Physiology and Graduate Institute of Basic Medical Science, School of Medicine, College of Medicine, China Medical University, No.91 Hsueh-Shih Rd., Taichung 404, Taiwan; E-Mails: ywc@mail.cmu.edu.tw (Y.-W.C.); tain_hui@hotmail.com (T.-H.L.); 9 School of Chinese Medicine, College of Chinese Medicine, China Medical University, No.91 Hsueh-Shih Rd., Taichung 404, Taiwan; E-Mail: sychang@mail.cmu.edu.tw

**Keywords:** mercuric compounds, pancreatic islets, oxidative stress, apoptosis

## Abstract

Mercury is a toxic heavy metal that is an environmental and industrial pollutant throughout the world. Mercury exposure leads to many physiopathological injuries in mammals. However, the precise toxicological effects of mercury on pancreatic islets *in vivo* are still unclear. Here, we investigated whether mercuric compounds can induce dysfunction and damage in the pancreatic islets of mice, as well as the possible mechanisms involved in this process. Mice were treated with methyl mercuric chloride (MeHgCl, 2 mg/kg) and mercuric chloride (HgCl_2_, 5 mg/kg) for more than 2 consecutive weeks. Our results showed that the blood glucose levels increased and plasma insulin secretions decreased in the mice as a consequence of their exposure. A significant number of TUNEL-positive cells were revealed in the islets of mice that were treated with mercury for 2 consecutive weeks, which was accompanied by changes in the expression of the mRNA of anti-apoptotic (*Bcl-2*, *Mcl-1*, and *Mdm-2*) and apoptotic (*p53*, *caspase-3*, and *caspase-7*) genes. Moreover, plasma malondialdehyde (MDA) levels increased significantly in the mice after treatment with mercuric compounds for 2 consecutive weeks, and the generation of reactive oxygen species (ROS) in the pancreatic islets also markedly increased. In addition, the mRNA expression of genes related to antioxidation, including *Nrf2*, *GPx*, and *NQO1*, were also significantly reduced in these islets. These results indicate that oxidative stress injuries that are induced by mercuric compounds can cause pancreatic islets dysfunction and apoptosis *in vivo*.

## 1. Introduction

Mercury, a toxic heavy metal and a widespread environmental pollutant, poses a serious health hazard [1,2]. Mercury is normally present in 3 forms-elemental mercury (Hg^0^), inorganic mercury (Hg^2+^ and Hg^+^), and organic mercury (methylmercury, MeHg)-all of which can produce varying degrees of toxic effects in many organs or systems. These effects include cardiovascular disease, endocrine system disruption, neurotoxicity, and immunotoxicity [3–5]. A previous study indicated that approximately 80% of mercury vapor (inorganic mercury) is inhaled through the lungs and then absorbed into the bloodstream, and remaining in the circulation for a long enough period to be distributed to other tissues. The organic form of mercury, MeHg, causes an irreversible neurotoxic disorder in mammals through biotransformation in the food chain, such as consumption of contaminated fish, seafood, and aquatic mammals [6,7]. The pancreatic islet cells destroyed and an increased incidence of diabetes mellitus (DM) was found in patients with Minamata disease (MeHg poisoning) in Japan [8,9] The study of Shigenaga [10] also found that repeated treatment of rats with MeHg induced a high blood glucose level that was accompanied by pancreatic islets injuries. Recently, Chen *et al.* [11,12] reported that mercuric compounds exposure can induce pancreatic β-cell dysfunction and death *in vitro*. However, the toxicological effects and possible mechanism by which mercuric compounds caused damage to the pancreatic islets *in vivo* remained to be clarified.

DM is part of a group of metabolic diseases that is characterized by hyperglycemia originating from defects of insulin secretion by the pancreatic β-cells and/or insulin action in the peripheral tissues. Many studies have reported that the death of pancreatic islet β-cells contributes to type 1 (insulin-dependent) diabetes, which is a prototype of organ-specific autoimmune diseases in which an immune-mediated inflammation results in the selective destruction and infiltration of islet β-cells, inhibits insulin secretion, and causes pancreatic β-cell death [13,14]. Some insults, such as lipoxygenases (expressed in human and rodent islets), can cause injury by inducing oxidative stress-regulated inflammatory damage and cell death in islet β-cells [15]. In addition, the production of reactive oxygen species (ROS) results in oxidative stress, which induces undesirable biological reactions and injuries to functional cells, including pancreatic islet β-cell dysfunction and apoptosis, that are caused by cytokines or autoimmune attack in type 1 DM. Pancreatic β-cells are reported to be vulnerable to oxidative stress damage [16,17]. Toxic metals, such as mercury and arsenic, can induce toxic effects via oxidative stress leading to apoptosis and pathophysiological injuries, which then cause to many disorders including DM [18–21]. Taken together, in this study, we sought to elucidate the toxicological effects induced by mercuric compounds (MeHg and mercuric chloride (HgCl_2_)) in the pancreatic islets of male mice (*in vivo* model) and to explore the hypothesis that mercuric compounds-induced oxidative stress damage leads to dysfunction and apoptosis in pancreatic islets. To examine these issues, we investigated the deleterious effects of exposure to MeHg (2 mg/kg/day) and HgCl_2_ (5 mg/kg/day) for 2 to 6 consecutive weeks in male mice by monitoring the changes in blood glucose, plasma insulin, and MDA levels, and by analyzing the Hg concentration of mouse whole blood samples. Moreover, we examined whether exposure to mercuric compounds could induce apoptosis and ROS generation while altering apoptotic- and antioxidant-related gene expression in the islets of treated mice at the end of 2 weeks.

## 2. Results and Discussion

### 2.1. Effects of Mercuric Compounds on Blood Glucose Regulation and Plasma Insulin Levels in Mice

**Figure 1:**
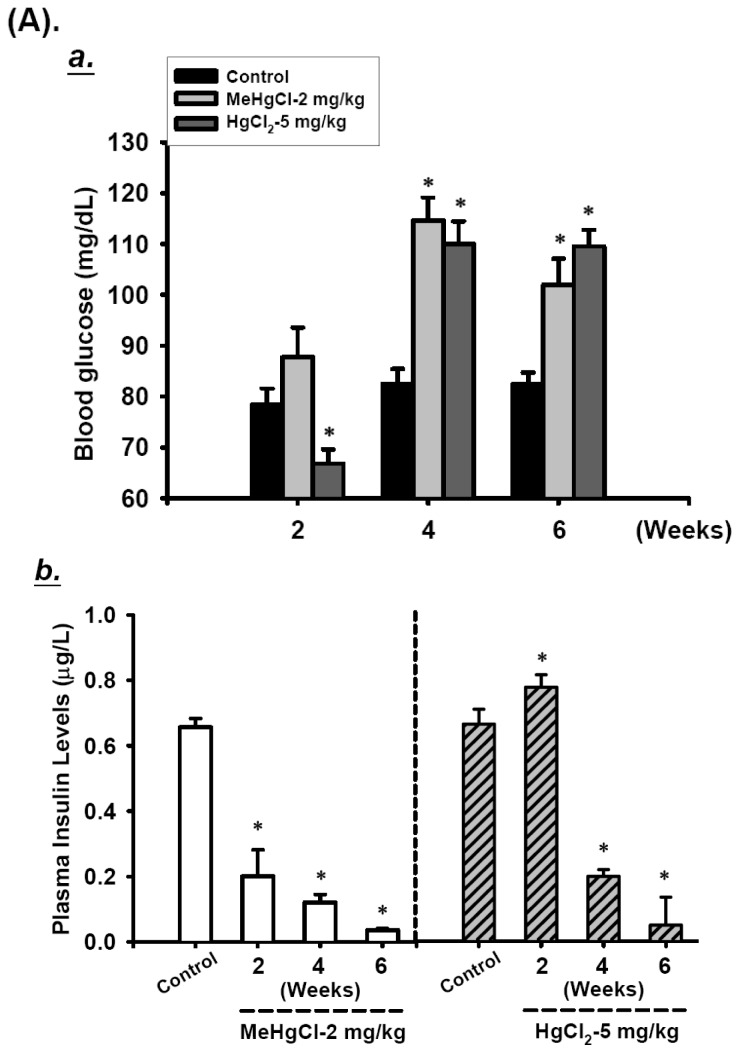

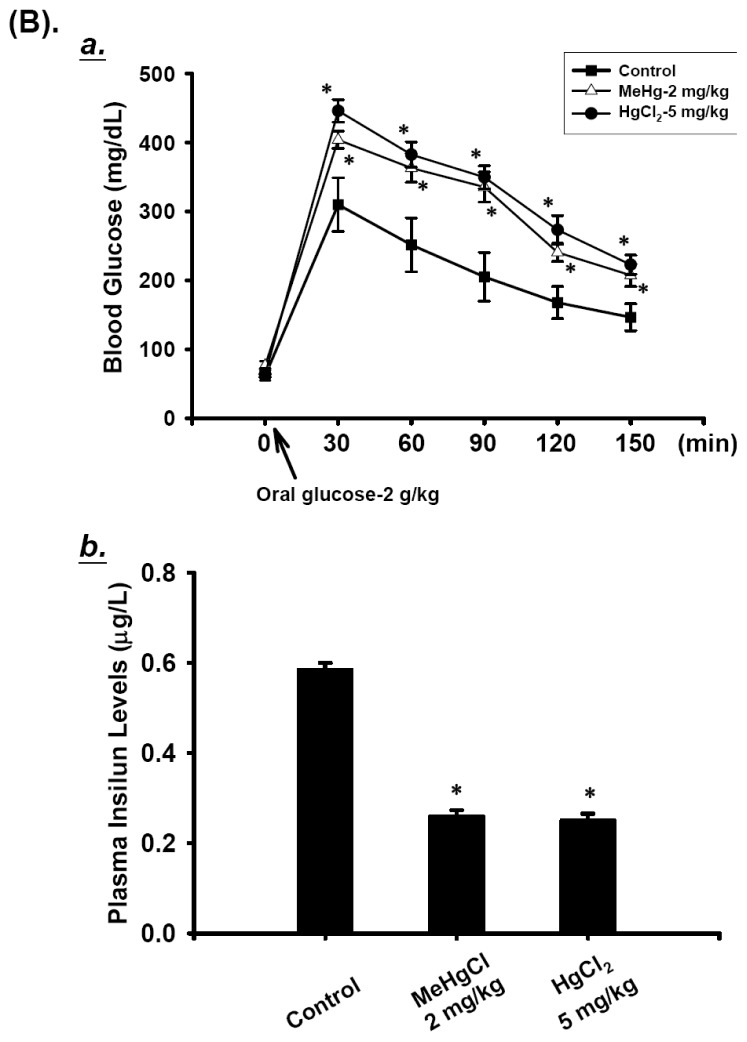
Effects of mercuric compounds on the regulation of blood glucose and plasma insulin levels in mice. (**A**) Mice were gavaged with 2 mg/kg/day MeHgCl or 5 mg/kg/day HgCl_2_ for 6 consecutive weeks. Fasting blood glucose was determined by SureStep blood glucose meter (**A**,**a**), and the plasma insulin levels were analyzed by insulin enzyme-linked immunosorbent assay (ELISA) assay kit (**A**,**b**) at 2, 4, and 6 weeks. (**B**) Oral glucose tolerance and insulin in fasting mice were determined as described in the Materials and Methods section. Oral glucose tolerance tests were carried out in mice given 2 mg/kg/day MeHgCl or 5 mg/kg/day HgCl_2_ for 2 consecutive weeks (**B**,**a**). Plasma insulin levels in mercuric compounds-treated mice after 2 g/kg glucose loading for 30 min were analyzed (**B**,**b**). All data are presented as means ± standard errors of the mean (SEM). (*n* = 16 mice for each group).* *p* < 0.05 compared with the control group.

To investigate the effects of mercuric compounds on *in vivo* pancreatic islet function, we monitored the changes in blood glucose and plasma insulin levels in MeHgCl or HgCl_2_-exposed mice. Fasting blood glucose levels in mice showed a marked increase and the plasma insulin levels decreased after 4 or 6 consecutive weeks of exposure to MeHgCl (2 mg/kg/day) or HgCl_2_ (5 mg/kg/day) as compared with the control group (Figure 1A). After 2 consecutive weeks of exposure to MeHgCl, it was showed a light, but not statistically significant, increase in blood glucose levels, but there was a remarkable decrease in plasma insulin levels. By contrast, mice exposed to HgCl_2_ for 2 consecutive weeks were showed a significant decrease in blood glucose levels and increased plasma insulin levels (Figure 1A). To confirm that exposure to mercuric compounds can cause islet damage resulting in blood glucose dysregulation, we used the oral glucose tolerance test (OGTT). As shown in Figure 1B, both MeHgCl and HgCl_2_-exposed mice revealed an elevation in glucose intolerance (Figure 1B,a), and it was also a marked decrease in plasma insulin after glucose loading for 30 min following 2 consecutive weeks of exposure. Moreover, the mercury levels in the whole blood of mice exposed to mercuric compounds over a 2- to 6- consecutive weeks period were significantly elevated (MeHgCl group: 4970.8 ± 38.8 μg/L, 14827.6 ± 1938.7 μg/L, and 27741.4 ± 6747.1 μg/L at 2, 4, and 6 weeks, respectively; HgCl_2_ group: 432.0 ± 111.2 μg/L, 683.4 ± 47.9 μg/L, and 865.8 ± 222.5 μg/L at 2, 4, and 6 weeks, respectively; age-matched control group ranged from 2.4 ± 0.3 μg/L to 3.0 ± 0.5 μg/L) (Table 1). These results suggest that treatment with MeHgCl or HgCl_2_ destroys pancreatic islet function in mice.

### 2.2. Mercuric Compounds Caused Apoptosis in the Pancreatic Islets of Exposed Mice

**Figure 2:**
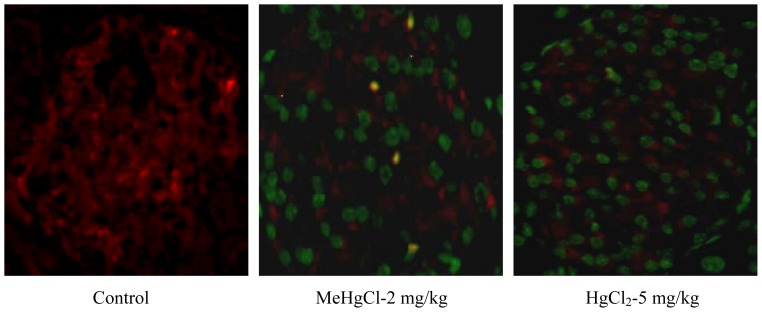
Immunofluorescence analysis of islet apoptosis induction in mercuric compounds-exposed mice. Mice were gavaged with 2 mg/kg/day MeHgCl or 5 mg/kg/day HgCl_2_ for 2 consecutive weeks, and dual immunofluorescence staining of islet using anti-insulin (red) and TUNEL (green) was performed as described in the Materials and Methods section (400×).

**Figure 3:**
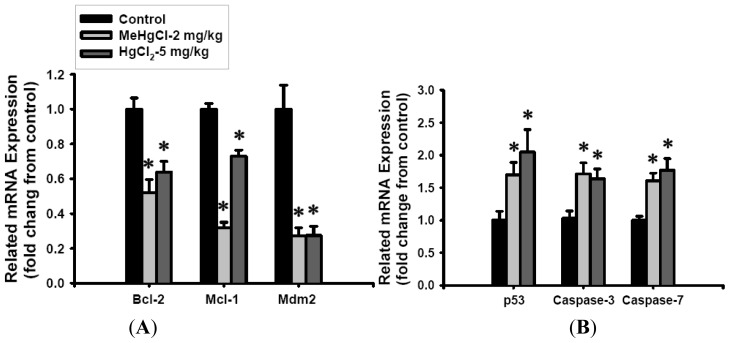
Mercuric compounds treatment regulated apoptotic related gene expression in the islets of mice. Mice were gavaged with 2 mg/kg/day MeHgCl or 5 mg/kg/day HgCl_2_ for 2 consecutive weeks, and the expression of anti-apoptotic (*Bcl-2*, *Mcl-1*, *Mdm-2*) (**A**) and apoptotic (*p53*, *caspase-3* and *caspase-7*) (**B**) genes in the isolated islets were analyzed by real-time quantitative RT-PCR using SYBR Green. Target gene expression was normalized to β-actin, and the results are expressed as a fold change from the control. Results are expressed as mean ± SEM. (*n* = 8 mice for each group). * *p* < 0.05 as compared with control group.

To investigate whether mercuric compounds induce dysfunction of pancreatic islets via an apoptotic mechanism, we performed terminal deoxynucleotidyl transferase dUTP nick end labeling (TUNEL) and insulin dual staining, and were measured the expression of apoptosis-related genes (by real-time quantitative RT-PCR). As shown in Figure 2, the number of TUNEL-positive cells in the isolated islets of mice was significantly increased after exposure to MeHgCl (2 mg/kg/day) or HgCl_2_ (5 mg/kg/day) for 2 consecutive weeks, which only revealed a weak insulin immunoreactivity in comparison with the control group. In addition, the expression of *Bcl-2*, *Mcl-1*, and *Mdm-2* (anti-apoptotic genes) were showed an obvious decreased (Figure 3A), while that of *p53* (apoptotic gene) dramatically increased; these changes were accompanied by a marked up-regulation of *caspase-3* and *caspase-7* gene expression levels (approximately 1.5 to 2.0 fold; Figure 3B) in the isolated islets of mice exposed to MeHgCl or HgCl_2_. These results indicate that exposure to mercuric compounds *in vivo* can cause injury to pancreatic islets, leading to a pathophysiological state associated with apoptosis.

### 2.3. Exposure to Mercuric Compounds Induced Oxidative Stress Damage in the Pancreatic Islets

**Figure 4:**
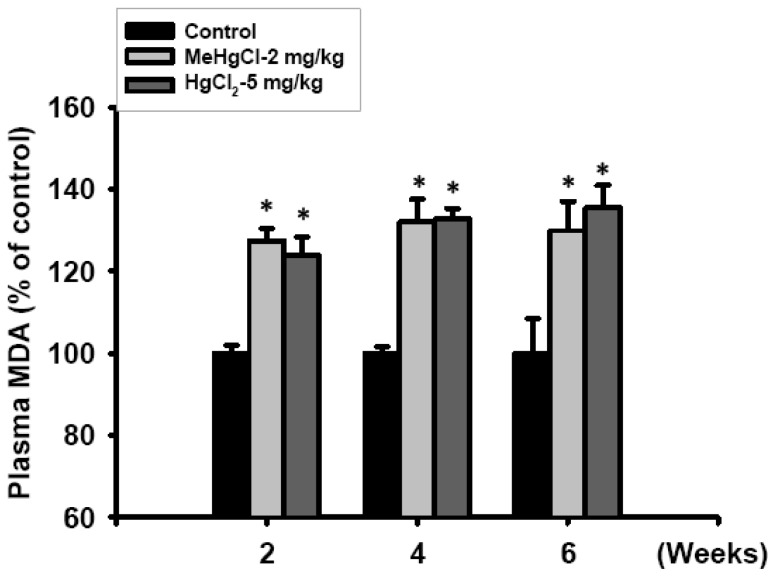
Effect of mercuric compounds on plasma lipid peroxidation (LPO) levels in mercuric compounds-exposed mice. Mice were gavaged with 2 mg/kg/day MeHgCl or 5 mg/kg/day HgCl_2_ for 2 to 6 consecutive weeks. Malondialdehyde (MDA) levels of the plasma were determined using the commercial manufacturer’s assay kit as described in the Materials and Methods section. All data are presented as means ± SEM. (*n* = 16 mice for each group). * *p* < 0.05 compared with control group.

**Figure 5:**
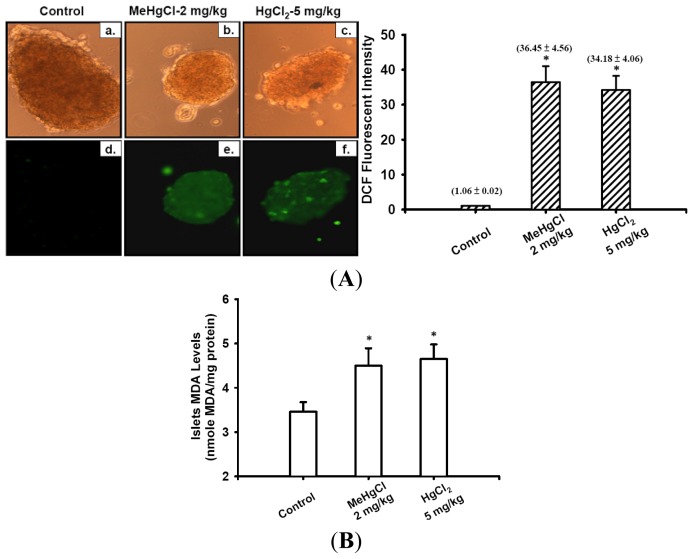
Mercuric compounds-triggered reactive oxygen species (ROS) generation in the islets of mice. Mice were treated (by oral gavaged) with 2 mg/kg/day MeHgCl or 5 mg/kg/day HgCl_2_ for 2 consecutive weeks, and the islets were isolated from mice. (**A**) The peroxide-sensitive fluorescent probe (DCFH-DA) was used to detect the ROS production in the islets. The upper panels were transmitted light images (**a**–**c**); staining with DTZ) and the lower panels were DCF fluorescence images (**d**–**f**) (200×). (**B**) Malondialdehyde (MDA) levels of the isolated islets were determined using the commercial manufacturer’s assay kit as described in the Materials and Methods section. Data in B are presented as means ± SEM. (*n* = 16 mice for each group). * *p* < 0.05 compared with control group.

To further explore the involvement of oxidative stress damage in the mechanism underlying the toxicological effects induced by mercuric compounds in the islets of mice, we analyzed lipid peroxidation (LPO) production (as an indicator of oxidative stress damage) in the plasma and ROS generation in the islets of exposed mice. After the mice treated with MeHgCl (2 mg/kg/day) or HgCl_2_ (5 mg/kg/day) for 2 to 6 consecutive weeks, the plasma MDA levels were significantly increased at 2 weeks and continued to increase at 4 and 6 weeks (Figure 4). In addition, the results of 2′,7′-dichlorofluorescein (DCF) fluorescence probe intensity (as an indicator of ROS formation) and LPO assay also revealed that the intracellular ROS production (Figure 5A) and MDA levels (Figure 5B) in the isolated islets markedly increased after the mice were treated with MeHgCl or HgCl_2_ for 2 consecutive weeks.

**Figure 6:**
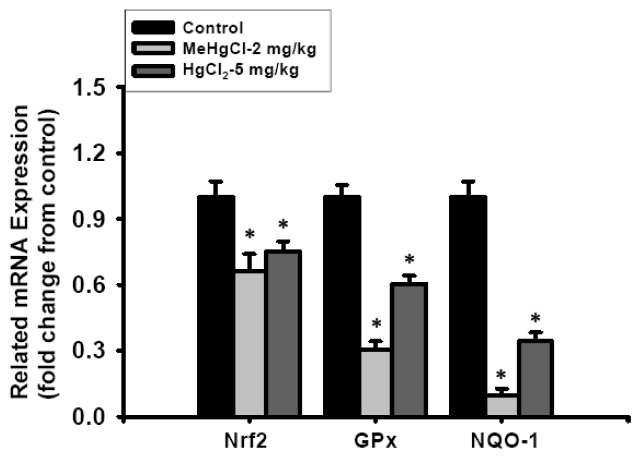
Related anti-oxidant gene expression in the isolated islets of mice exposed (by gavage) to 2 mg/kg/day MeHgCl or 5 mg/kg/day HgCl_2_ for 2 consecutive weeks. The expression of *Nrf2*, *GPx*, and *NQO1* genes was determined by real-time quantitative RT-PCR using SYBR Green. Target gene expression was normalized to β-actin, and the results are expressed as a fold change from the control. Results are expressed as mean ± SEM. (*n* = 8 mice for each group). * *p* < 0.05 as compared with control group.

Furthermore, we analyzed the mRNA expression levels of *Nrf2*, *GP_X_*, and *NQO1*, which play an important role in the antioxidant system. A significant decrease in the expression of *Nrf2*, *GP_X_*, and *NQO1* was revealed in the isolated islets of mice exposed to MeHgCl (2 mg/kg/day) or HgCl_2_ (5 mg/kg/day) for 2 consecutive weeks (Figure 6). These results indicate that exposure to mercuric compounds induces oxidative stress injury in islets *in vivo*.

### 2.4. Discussion

Many *in vivo* studies have reported that exposure to high doses of mercury (4–40 mg/L in drinking water or 0.2–2 mg/kg/day for more than 7 consecutive days) can cause severe neuropathological injuries and neurophysiological disorders [22,23]. Recently, the growing studies have also shown that the toxicological effects of MeHgCl (2–26 mg/kg/day) or HgCl_2_ (5–10 mg/kg/day) induced by long-term exposure within the cerebral cortex, liver, kidney, and lung of experimental animals were accompanied by a significant production of ROS [24–27]. ROS, which include superoxide anion, hydrogen peroxide, and hydroxyl radicals, are highly reactive and can damage cell structure and function [28]. Many factors, such as ionizing radiation, xenobiotics, and toxic metals can promote ROS generation, which triggers cell death and implicates in the development of various disorders [19,20]. Mercury induces toxic effects by causing oxidative stress from ROS production, which oxidizes the membrane lipids of cells and causes the alteration of cellular function, and eventually results in cell death and pathophysiological injuries in mammals [19–21,26]. Moreover, it has been reported that oxidative stress plays a crucial role in inducing pancreatic islet β-cell injuries and the pathogenesis of DM, probably as a result of excessive levels of mitochondrial ROS production and the presence of fewer antioxidant enzymes in pancreatic β-cells [29,30]. For these reasons, it was supported that oxidative stress might contribute to the induction of the pancreatic islet injuries resulting from mercury intoxication. Recently, Chen *et al.* [11,12] reported that treatment with mercuric compounds can induce dysfunction and cell death in a pancreatic β-cell-derived cell line; the role of ROS in the toxicological effects induced by mercury on the pancreatic islets (*in vivo*), however, has not been understood. In this study, our results showed that exposure to MeHgCl (2 mg/kg/day) or HgCl_2_ (5 mg/kg/day) for more than 2 consecutive weeks caused a significant impairment in blood glucose regulation and decreased plasma insulin levels in mice, which was accompanied by a marked accumulation of mercury in the whole blood. In addition, the significant increase in the number of TUNEL-positive cells and changes to the expression of apoptosis-related genes in the islets of mice exposed to mercury were also revealed, that was along with an increase in plasma MDA levels, the induction of ROS generation, and the decrease in antioxidant-related mRNA expressions. Therefore, our results indicate that mercury causes oxidative stress-induced apoptosis in pancreatic islet cells, leading to deleterious effects on blood glucose regulation *in vivo*.

Our results in this study found that a significant increase in blood glucose and a decrease in plasma insulin levels were showed after treatment mice with MeHgCl (at 2 to 6 consecutive weeks) or HgCl_2_ (at 4 to 6 consecutive weeks). It is also interesting to note that after the mice treated with HgCl_2_ for 2 consecutive weeks, the suppression of blood glucose was associated with an increase of plasma insulin levels; but a significant elevation of blood glucose intolerance and a decrease in plasma insulin after glucose loading were also revealed. This effect might be related to the started induction of islet cell apoptosis in early stage by HgCl_2_, leading to the rupture the insulin secretory vesicle membranes and resulting in insulin release. Furthermore, blood glucose homeostasis is generally regulated by skeletal muscle and adipose tissues. Growing studies have focused on the mechanism by which metal-mediated glucose transport contributes to metal-induced pathologies [31–33]. It has been reported that HgCl_2_ can increase the levels of glucose uptake and transport in the adipocytes [34,35]. However, some studies found the opposite results [36,37]. On the basis of these reasons, we suggest that HgCl_2_ may be responsible for the disturbance of blood glucose homeostasis; however, the detailed mechanisms of this disturbance still require future investigation.

To ascertain whether MeHgCl- and HgCl_2_-induced islet dysfunction and apoptosis *in vivo* was mediated by a stress-related mitochondrial pathway, we analyzed the mRNA expressions of *Bcl-2*, *Mcl-2*, *Mdm-2*, *p53*, *caspase-7*, and *caspase-3* in the islets of MeHgCl- or HgCl_2_-treated mice. Apoptosis, which is also known as programmed cell death, plays an important role in controlling the development of multicellular organisms and maintaining tissue homeostasis and in an increasing number of disease processes ranging from neurodegenerative diseases to the development of DM [30,38]. Caspases are cysteine aspartate proteases, which are represented the hallmark of the apoptotic process. Activation of the mitochondria (intrinsic)-regulated apoptosis pathway results in the activation of the BH3-only members of the Bcl-2 family (*i.e.*, Bim, Bid, Bad, Puma, Noxa, Hrk, Bik, and Bmf) to initiate apoptosis signaling by binding to Bcl-2-like prosurvival proteins (*i.e.*, Bcl-2, Bcl-xL, Bcl-w, and Mcl-1). The downstream apoptosis effector Bax or the Bax-related effector Bak is subsequently released, which induces a decrease in the mitochondrial outer membrane potential and the release of cytochrome *c* from mitochondria into the cytosol, leading to the activation of the caspase family of proteins [39,40]. In addition, the enhancement of Bak interaction with p53 also stimulates apoptotic effects [41]. Mdm2, an important negative regulator of the p53 tumor suppressor, binds to p53 and inhibits p53-mediated transactivation. Increased levels of mdm2 can inactivate the apoptotic and cell cycle arrest functions of p53 and regulate cell proliferation [42]. Recent studies have shown that the involvement of these apoptosis-related gene alterations in response to toxic metals (such as mercury and arsenic) can induce apoptosis [21,43,44]. Here, we found that the expression of anti-apoptosis-related mRNAs, including *Bcl-2*, *Mcl-*1, and *mdm-2*, were significantly decreased in the islets of mice treated with MeHgCl and HgCl_2_ for 2 consecutive weeks. Furthermore, the expression of apoptosis-related mRNAs, including *p53*, *caspase-3*, and *caspase-7* were dramatically increased. These effects were accompanied by a significant increase in TUNEL-positive cells in the islets of mercuric compounds-treated mice.

Oxidative stress, induced by toxic metals (including mercury), plays an important role in apoptosis and pathological injuries, which are accompanied by damage to antioxidant enzymes [20,21,44]. The Nrf2 pathway has been implicated in the cell’s response to pro-oxidant and electrophilic damage. Following the electrophilic attack, Nrf2 is released from Keap1-Nrf2 complex and translocated from the cytosol to the nucleus. Subsequently, the Nrf2 binds to antioxidant and electrophile response elements, and increases in the expression of many other antioxidant genes and proteins, such as heme oxygenase-1 (HO-1), glutathione S-transferase A2 (GSTA2), thioredoxin reductase, and NAD(P)H quinone oxidoreductase (NQO1) [45–47]. Enhancing these ROS-scavenging capacities is important in maintaining cellular redox homeostasis and decreasing oxidative stress [48]. Recently, Ni *et al.* [49] has reported that a knockdown of Nrf2 greatly increased microglial cell death during MeHg exposure. Furthermore, the encoded protein NQO1 is a member of the NAD(P)H dehydrogenase (quinone) family, which forms homodimers and reduces quinones to hydroquinones to prevent ROS production from the reduction of an electron in the quinone. Thus, NQO1 plays a classical direct antioxidant role in the detoxification of ROS, which can be induced by the overproduction of free radicals from toxic chemicals- induced oxidative stress [48,50,51]. Moreover, glutathione peroxidase (GPx) is an important antioxidant enzyme that catalyzes the reduction of hydrogen peroxide (H_2_O_2_) [52]. However, whether mercury-induced ROS production leading to apoptosis accompanied with the decrease in the gene expression level of antioxidants (including: *Nrf2*, *GPx*, and *NQO1*) in the islets of mercury-exposed mice remained to be clarified. The present work showed that DCF fluorescence intensity and MDA levels in the islets of mice treated with MeHgCl or HgCl_2_ for 2 consecutive weeks were dramatically increased. Furthermore, the mRNA expression of *Nrf2*, *GPx*, and *NQO1* were remarkably decreased in the islets of mice treated with mercuric compounds. These results implicate that mercury-induced oxidative stress plays a key role in pancreatic islets dysfunction and death *in vivo*.

## 3. Experimental Section

### 3.1. Animal Preparation and Study Design

Male ICR mice (6 weeks old, 20–25 g) were obtained from the animal center of the BioLASCO Taiwan Co., Ltd. (Taipei, Taiwan). Experimental protocols were approved by the Institutional Animal Care and Use Committee (IACUC) and the care and use of laboratory animals were conducted in accordance with the guidelines of the Animal Research Committee of China Medical University. The mice were housed in groups of six per cage under standard laboratory conditions at a constant temperature (23 ± 2 °C), 50% ± 20% relative humidity, and given a solid diet and tap water available *ad libidum*, with 12 h:12 h light-dark cycles. The mice were acclimatized to the laboratory conditions prior to the experiments and all tests were carried out between 8:00 AM and 05:00 PM. The mice were randomly divided into 3 groups: MeHgCl (2 mg/kg/day), HgCl_2_ (5 mg/kg/day), and age-matched control (distilled water only) that were orally gavaged for 2 to 6 consecutive weeks. At each time point (2, 4, and 6 weeks; *n* = 16 mice for each group), the whole blood samples were collected from an eyehole vessel (under anesthesia), and whole blood mercury concentrations were detected. Morover, the whole blood samples were centrifuged at 3000*g* for 10 min, and plasma was obtained, and insulin and LPO levels were assayed immediately.

Prior to pancreatic islet isolation and purification, the mice were treated with or without mercuric compounds for 2 consecutive weeks (*n* = 8 mice for each group) and then sacrificed by decapitation under pentobarbital anesthesia (80 mg/kg, intra-peritoneal (i.p.)). The pancreas was quickly removed and the islets were isolated. After islets purification, the ROS levels, MDA levels, and apoptosis- and antioxidant-related genes expression were analyzed.

### 3.2. Blood Glucose Measurement and Oral Glucose Tolerance Test (OGTT)

Mice were treated with MeHgCl (2 mg/kg/day), HgCl_2_ (5 mg/kg/day), or distilled water (age-matched control group; *n* = 16 mice for each group) for 2 to 6 consecutive weeks. Blood samples were collected from mouse eyehole after an overnight fast, and blood glucose levels were measured using an OneTouch^®^ SureStep^®^ blood glucose meter (Lifescan, Milpitas, CA, USA). Oral glucose tolerance test (OGTT) was performed as previously detailed [43]. Mice were fed with D-glucose by gavage after an overnight fast. Blood was collected (from an eyehole) before treatment and 30, 60, 90, 120, and 150 min after glucose administration.

### 3.3. Plasma Insulin Level Detection

To measure the plasma insulin concentration, the whole blood of mice treated with MeHgCl (2 mg/kg/day), HgCl_2_ (5 mg/kg/day), or distilled water (age-matched control group; *n* = 16 mice for each group) for 2 to 6 consecutive weeks was collected and centrifuged at 3000*g* for 10 min to obtain plasma. Aliquots of samples were then subjected to insulin antiserum immunoassay according to the manufacturer’s instructions (Mercodia, Uppsala, Sweden).

### 3.4. Determination of Mercury Concentrations

To determine the Hg concentrations, 300 mg of whole blood from each mouse was placed in a 15 mL polyethylene tube, and 0.5–1 mL of a 3:1 mixture of hydrochloric acid (35%) and nitric acid (70%) was added. The tubes were capped and allowed to stand overnight in a 50 °C oven. After cooling, a suitable dilution buffer (0.3% nitric acid and 0.1% Triton X-100 in distilled water) was added to the digested material, and the total mercury content was determined by Inductively Coupled Plasma Mass Spectrometry (ICP-MS). The detection limit for mercury was ~0.1 ppb (μg/L).

### 3.5. TUNEL and Insulin Double Staining

Mice were treated with MeHgCl (2 mg/kg/day), HgCl_2_ (5 mg/kg/day), or distilled water (age-matched control group) for 2 consecutive weeks, and their pancreases were isolated and fixed in 10% formaldehyde in PBS. To examine apoptosis in islets, TUNEL and insulin (to identify β-cells) double immunostaining was performed on 5-μm pancreas sections (onparaffin slide). After deparaffinization and rehydration, TUNEL staining was performed using a fluorometric transferase-mediated TUNEL assay kit (Promega Corporation, Madison, WI, USA) by following the manufacturer’s procedure. Following the TUNEL stain, the slide was rinsed in phosphate-buffer saline (PBS) and incubated with a rabbit polyclonal IgG anti-insulin antibody (Santa Cruz Biotechnology, Inc., CA, USA) for 1 h at room temperature. The slide was then washed four times with PBS and incubated with the secondary antibody labeled with Cy3 (Millipore Corporation, Billerica, MA, USA) for 1 h. After being washed twice with PBS, the slide was observed with a Leica DMIL inverted fluorescence microscope equipped with a charge-coupled device camera (400× magnification).

### 3.6. Lipid Peroxidation Detection

The formation of MDA, a substance produced during lipid peroxidation, was determined using a commercial LPO assay kit (Calbiochem, San Diego, CA, USA) according to the manufacturer’s instructions. Briefly, the isolated islets were homogenized separately in ice-cold 20 mM Tris-HCl buffer (pH 7.4), and then the homogenized samples were assayed immediately. Equal volumes of plasma and islet homogenates (*n* = 16 mice for each group) were added to 3.25 volumes of diluted R1 reagent (10.3 mM *N*-methyl-2-phenylindole in acetonitrile). After mixing, the mixture was added to a 0.75 volumes of 37% HCl and was then incubated at 45 °C for 60 min. After cooling, the absorbance of the clear supernatant was subjected to an enzyme-linked immunosorbent assay (ELISA) microplate and read at 586 nm. The linearity of the standard curve was confirmed with 0, 1, 2.5, 5, 10, 20, and 40 μM of MDA standard (1,1,3,3-tetramethoxypropane in Tris-HCl). The protein concentration was determined using a bicinchoninic acid protein assay kit with an absorption band of 570 nm. (Pierce, Rockford, IL, USA).

### 3.7. Pancreatic Islet Isolation and Purification Procedure

Isolation of the mouse islet of Langerhans was performed as previously described [53,54]. In brief, collagenase, prepared in Hank’s balanced salt solution with 25 mM Hepes, was infused into the main bile duct of each mouse with a 30-gauge needle connected to a 10 cc syringe. The whole pancreas was collected and digested at 37 °C. The islets were obtained using a Ficoll density gradient of 1.069–1.096 g/mL. The number of islets was counted by staining samples with dithizone (DTZ), and the islet equivalent (IEQ) range was 75–150 μm (where 1 IEQ is equivalent to an islet with a diameter of 150 μm). The islets were cultured in RPMI 1640 medium containing fetal bovine serum (10%), penicillin-streptomycin (100 unit/mL), L-glutamate (2 mM), and Hepes (25 mM).

### 3.8. Real-Time Quantitative Reverse-Transcribed Polymerase Chain Reaction (RT-PCR) Analysis

The expression of related genes was evaluated by the real-time quantitative RT-PCR as previously described [21,44]. Briefly, intracellular total RNA was extracted from 300 IEQ islets of each mouse (*n* = 16 mice for each group) using RN easy kits (Qiagen) according to the manufacturer’s instructions. Samples were heated to 90 °C for 5 min to remove any secondary structures, and then placed immediately on ice. Samples were reverse transcribed into cDNA using the AMV RTase system (Promega Corporation, Pty. Ltd., Madison, Wisconsin, USA) according to the manufacturer’s instructions. Each sample (2 μL) was tested with the Sybr Green Real-time PCR reagent (Invitrogen, Grand Island, NY, USA) using mouse-specific primers ((1) *Bcl-2*, *Mcl-1*, *p53*, *Caspase-3*, *Caspase-7*, glutathione peroxidase (*GPx*), NAD(P)H quinone oxidoreductase (*NQO-1*), and *β-Actin* as described in Lu *et al*. [21] and Yen *et al*. [44]; (2) murine double minute 2 (*mdm2*): forward: 5′-GGAGCGCAA AACGACACTTACA-3′ and Reverse: 5′-CTCGCTGCTGCTGCTGCTAC-3′ [55]; (3) nuclear factor erythroid-derived 2-related factor 2 (*Nrf2*) : forward: 5′-TGAAGCTCAGCTCGCATTGATCC-3′ and Reverse: 5′-AAGATACAAGGTGCTGAGCCGCC-3′ [56]) in a 25 μL reaction volume, and amplification and real-time fluorescence detection were performed using the ABI StepOnePlus sequence detection system (PE, Applied Biosystems, Carlsbad, CA, USA). The cycling conditions were as follows: 2 min at 50 °C, 10 min at 95 °C, 40 cycles of 92 °C for 30 s, and 1 min at 60 °C. Real-time fluorescence detection was performed during the 60 °C annealing/extension step of each cycle. A melt-curve analysis was performed on each primer set to ensure that no primer dimers or nonspecific amplification was present under the optimized cycling conditions. Data analysis was performed using StepOne™ software (Version 2.1, Applied Biosystems, Carlsbad, CA, USA, 2008). All amplification curves were analyzed with a normalized reporter (*R*_n_: the ratio of the fluorescence emission intensity to the fluorescence signal of the passive reference dye) threshold of 0.2 to obtain the *C*_T_ values (threshold cycle). The reference control genes were measured with four replicates from each PCR run, and the *C*_T_ average was used for relative quantification analyses (the relative quantification method using real-time PCR efficiencies [57]). TF expression data were normalized by subtracting the mean of the reference gene *C*_T_ values from their *C*_T_ values (Δ*C*_T_). The fold change value was calculated using the expression 2^−ΔΔ^*^C^*_T_, where ΔΔ*C*_T_ represents Δ*C*_T-condition of interest_ − Δ*C*_T-control_. Prior to conducting statistical analyses, the fold change from the mean of the control group was calculated for each individual sample.

### 3.9. Detection of Intracellular ROS in Islets

Mice were treated with MeHgCl (2 mg/kg/day), HgCl_2_ (5 mg/kg/day), or distilled water (age-matched control group) for 2 consecutive weeks, and the islets were then isolated. After 15 min of incubation of 2′,7′-dichlorfluorescein diacetate (DCFH-DA), the islets were washed twice in PBS and the images were captured using a Leica DMIL inverted fluorescence microscope equipped with a charge-coupled device camera (with 200× magnification).

### 3.10. Statistical Analyses

Data are presented as means ± standard errors of the mean (SEM). Significant differences were evaluated using Student’s *t*-test. When more than one group was compared with the control, the significance was evaluated according to a one-way ANOVA, and the Duncan’s post hoc test was applied to identify group differences. The *p* value less than 0.05 was considered to be significant. The statistical package SPSS, version 11.0 for Windows (SPSS Inc., Chicago, IL, USA, 2001) was used for the statistical analyses.

## 4. Conclusions

Collectively, the present *in vivo* results provide evidence that mercuric compounds (MeHgCl and HgCl_2_) are capable of causing pancreatic islet dysfunction (elevated blood glucose levels and decreased plasma insulin secretion) and apoptosis (decreased anti-apoptotic (*Bcl-2*, *Mcl-1*, and *mdm-2*) and increased apoptotic (*p53*, *caspase-3*, and *caspase-7*) related gene expressions) in treated mice. More importantly, this study has demonstrated that mercuric compounds induce pancreatic islet apoptosis *in vivo* through ROS generation, which leads to the destruction of antioxidant enzyme function (decreased the mRNA expressions of *Nrf2*, *GPx*, and *NQO1*). These observations further clarify that mercuric compounds-induced oxidative stress injuries cause pancreatic islet dysfunction and apoptosis *in vivo*.

## Figures and Tables

**Table 1:** Whole blood mercury levels in mercuric compounds-exposed mice.

Weeks	Group		

	Control	MeHgCl-2 (mg/kg)	HgCl_2_-5 (mg/kg)
2	2.4 ± 0.3	4970.8 ± 38.8 *	432.0 ± 111.2 *
4	2.6 ± 0.4	14827.5 ± 1938.7 *	683.4 ± 47.9 *
6	3.0 ± 0.5	27741.4 ± 6747.1 *	865.8 ± 222.5 *

1. Hg content was expressed as μg/L; 2. Data are expressed as mean ± SEM (*n* = 16 mice for each group).

* *p* < 0.05 as compared with control group.
